# Quantitative Magnetic Resonance Angiography Fails to Screen In-Stent Restenosis but Predicts Good Long-Term Outcome

**DOI:** 10.7759/cureus.15395

**Published:** 2021-06-02

**Authors:** Ahmad Ballout, Julia R Schneider, Jeffrey Katz

**Affiliations:** 1 Neurology, North Shore University Hospital/Hofstra Northwell School of Medicine, Manhasset, USA

**Keywords:** wingspan stent, in-stent restenosis, quantitative magnetic resonance angiography, atherosclerosis, vertebrobasilar circulation

## Abstract

Wingspan stent in the setting of symptomatic intracranial atherosclerotic disease (ICAD) has shown to be associated with in-stent restenosis (ISR). Conventional angiography is typically used to detect ISR, but quantitative magnetic resonance angiography (QMRA), a non-invasive measure of blood hemodynamics, has also been used to screen for ISR. This report highlights a case where QMRA failed to screen for ISR in a patient who received a Wingspan stent for symptomatic intracranial vertebral artery stenosis but predicted good long-term outcome. The patient remained asymptomatic and had robust vertebrobasilar flow at long-term follow-up despite developing ISR.

## Introduction

The use of the Wingspan stent for symptomatic intracranial atherosclerotic disease (ICAD) is associated with alarmingly high rates (29.7%) of in-stent restenosis (ISR), yet most patients will remain asymptomatic (76%) at six-month to one-year follow-up [1]. Screening for ISR is primarily done using digital subtraction angiography since non-invasive imaging modalities are obscured by stent-related artifact. Quantitative magnetic resonance angiography (QMRA), a tool used to measure blood hemodynamics, has been suggested as an alternative and/or adjunct tool to screen for ISR, with the added benefit of possibly risk-stratifying patients based on flow. The hemodynamic insight gained from QMRA can guide treatment given the uncertain therapy for this select population. In this case, we used QMRA over a five-year period to assess vertebrobasilar hemodynamics after Wingspan stent placement and subsequent ISR. This case supports the use of QMRA as a supplementary tool rather than a substitute for catheter angiography.

## Case presentation

**Figure 1:**
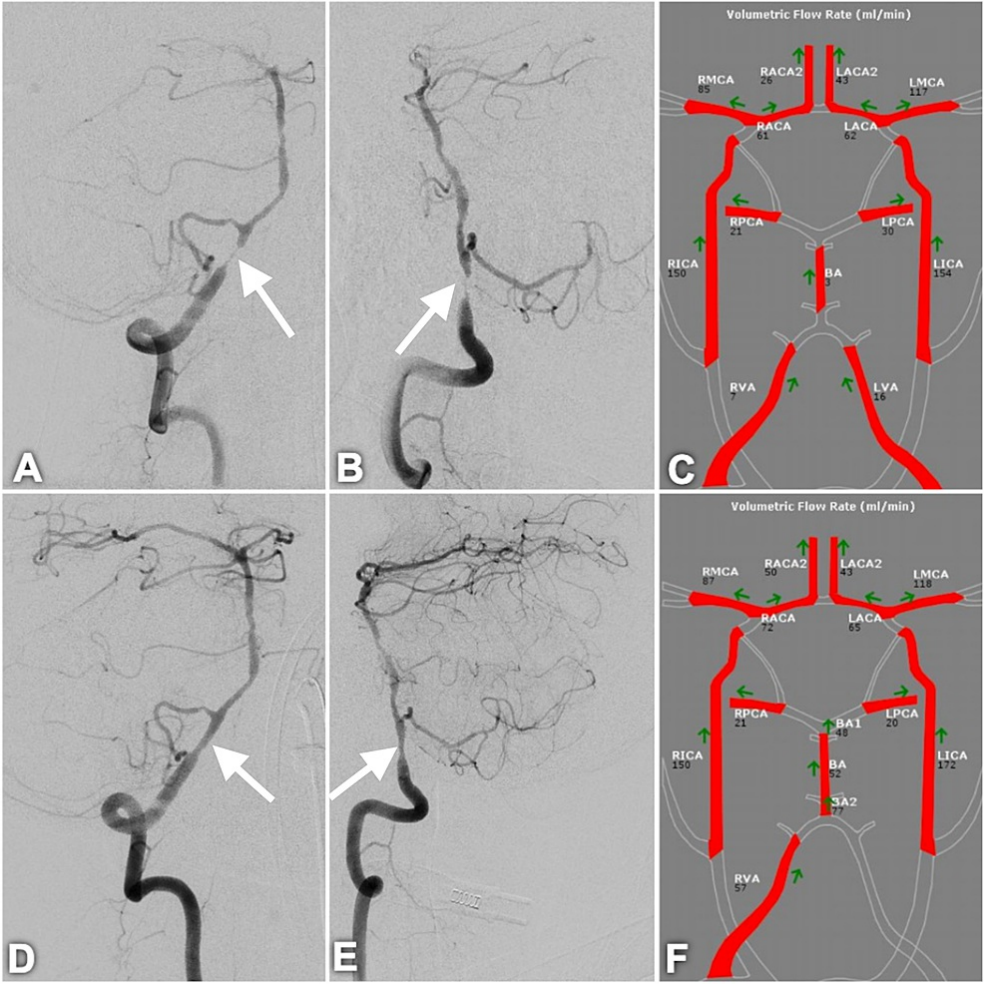
Pre- and post-Wingspan stent cerebral angiography and quantitative magnetic resonance angiography Pre-stent cerebral angiogram demonstrating severe intradural right vertebral artery stenosis (white arrow) (anteroposterior view, A; lateral view, B). Pre-stent quantitative magnetic resonance angiography demonstrating poor flow through the basilar and both vertebral arteries (C). Cerebral angiography status post-balloon angioplasty and Wingspan stent placement of the intradural right vertebral artery (white arrow) (anteroposterior view, D; lateral view, E). Quantitative magnetic resonance angiography performed at three-month post-operative follow-up demonstrating improved flow through the vertebrobasilar system (F).

A 60-year-old male with a past medical history of hypertension, diabetes, hyperlipidemia, peripheral vascular disease, end-stage renal disease on hemodialysis, and chronic obstructive pulmonary disease initially presented with dizziness, vomiting, and vertigo. He was not on anti-platelet therapy at the time. His MRI demonstrated multifocal infarcts in the posterior circulation including both cerebellar hemispheres, right pons, and the left thalamus. Magnetic resonance angiography (MRA) of the head and neck demonstrated a small left vertebral artery that ends in the posterior inferior cerebellar artery (PICA) and severe stenosis of the intracranial right vertebral artery. During his hospitalization, he was pressure-dependent with alteration in mental status noted during hemodialysis and at a systolic blood pressure of <160 mmHg. Repeat MRI of the brain demonstrated progressive infarcts in the pons, both occipital lobes, and both thalami. QRMA was performed, which showed severely diminished flow in the basilar artery. Based on this, the patient underwent emergent right vertebral artery balloon angioplasty and Wingspan stent placement, though a 35% residual stenosis remained by post-treatment angiography. He was started on Aspirin, Plavix, and statin therapy. At three-month follow-up, baseline QMRA demonstrated improved flow in the right vertebral artery (57 cc/min) and basilar artery (77 cc/min proximal, 52 cc/min mid, 46 cc/min distal-basilar artery) (Figures 1A-1F).

**Figure 2:**
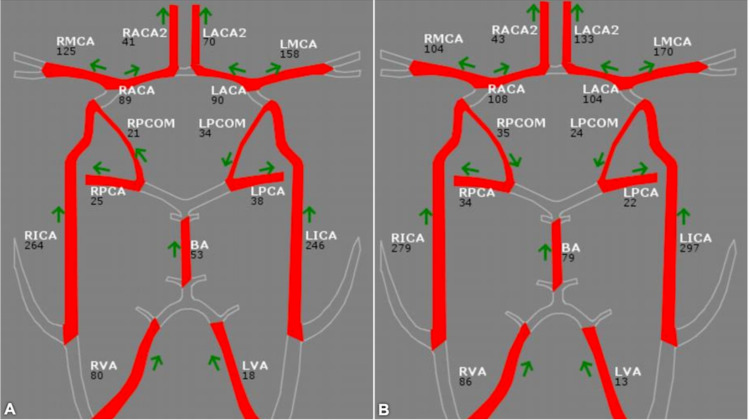
Follow-up quantitative magnetic resonance angiography Serial quantitative magnetic resonance angiography at 16-month follow-up (A) and 26-month follow-up (B) demonstrating robust flow through the vertebrobasilar system.

Right vertebral artery angiography at one-year follow-up demonstrated severe non-flow limiting ISR with preserved anterograde filling of the basilar artery. Given the ISR, he was continued on Aspirin, Plavix, and high-dose statin therapy. QMRA demonstrated normal flow throughout the vertebrobasilar system at the time of the ISR discovery and remained robust at two-year follow-up (Figures 2A, 2B). The patient remained asymptomatic without clinical episodes of vertebrobasilar insufficiency.

**Figure 3:**
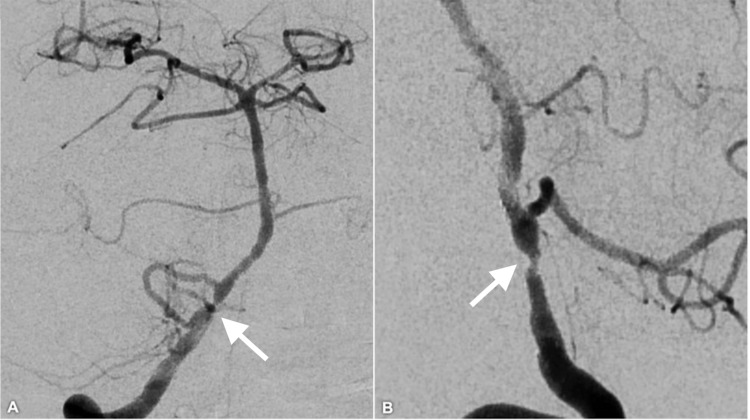
Conventional angiogram two years post-stent placement Conventional angiogram two years post-stent placement demonstrating persistent in-stent restenosis (white arrow) without anterograde flow-limitation (anteroposterior view, A; lateral view, B).

Conventional angiogram at two-year follow-up demonstrated unchanged ISR. The patient remained asymptomatic at five-year follow-up (Figures 3A, 3B).

## Discussion

This report highlights a unique case where QMRA accurately predicted a good outcome at five-year follow-up but failed to detect ISR. This differs from several case series that have demonstrated sensitivity rates as high as 100% when using QMRA in screening angiographic ISR [2,3]. In a case series involving 12 patients who underwent intracranial stenting for atherosclerosis, all six patients who developed angiographic ISR had an analogous decrease in flow of >25% by QMRA. Likewise, all six patients who did not have angiographic ISR had stable flow on QMRA [3].

Although the relationship between flow and the degree of vessel stenosis is non-linear, there is likely a point of "critical stenosis" that results in a significant flow reduction. This is widely thought to occur at approximately 70% arterial stenosis, though it can vary considerably depending on collateral flow. In a case series of nine patients who underwent Wingspan stent placement, a moderate correlation between flow and degree of stenosis was found using QMRA pre- and post-stenting (r = -0.670; p = 0.002) [4]. Our patient was clearly above the critical threshold pre-stent and likely below this threshold at the time of ISR. This may explain the robust flow in the face of ISR.

Using QMRA to risk-stratify patients with intracranial atherosclerosis has gained traction after the VERiTAS study showed a three times greater rate of recurrent stroke in a low-flow state compared to a normal-flow state [5]. The cohort of patients in VERiTAS was medically managed, and, therefore, the extrapolation of these data to the setting of ISR remains uncertain. In the case above [3], one of three patients with >25% decrement in flow was found to have recurrent strokes. Similarly, two of three patients with >50% decrement in flow had recurrent strokes. Our patient remained asymptomatic at five-year follow-up.

A major limitation of this case report is the lack of an immediate post-operative QMRA. The first post-treatment QMRA was performed at three-month follow-up, leaving a crucial time window where ISR may have theoretically developed and a decrement of flow - compared to the immediate post-operative hemodynamic outcome - potentially missed. Nonetheless, QMRA accurately predicted a good outcome in our patient based on flow status despite failing to screen positive for ISR because the ISR was sub-critical and not flow-limiting. This case supports the surveillance use of QMRA as a complementary test to be used in conjunction with cerebral angiography. A large prospective study evaluating the utility of QMRA in detecting ISR and predicting outcome is needed. Theoretically, QMRA may prove to be the preferred method for following these patients since it is the flow disturbance of ISR that is likely the paramount predictive factor in terms of long-term clinical outcome in patients after endovascularly treated ICAD.

## Conclusions

This case highlights a patient who developed ISR after a vertebral artery stent with serial QMRA scans post-operatively that failed to screen for ISR but predicted a good five-year outcome. This supports the use of QMRA as a supplementary tool rather than a substitute for catheter angiography, as it predicted a good outcome despite missing ISR. There is likely a unique critical stenosis threshold that varies across patients. By using QMRA as the sole screening tool, one may miss ISR if the stenosis remains below this theoretic threshold. QMRA may help risk-stratify patients based on flow and guide treatment options and need for re-stenting.
